# "They Don’t Come With a Handbook": Exploring Design Opportunities for Supporting Parent-Child Interaction around Emotions in the Family Context

**DOI:** 10.1145/3637409

**Published:** 2024-04-26

**Authors:** Nikki Theofanopoulou, Alissa N. Antle, Petr Slovak

**Affiliations:** King’s College London, UK; Simon Fraser University, Canada; King’s College London, UK

**Keywords:** Human-centered computing, Empirical studies in HCI, Empirical studies in interaction design, Scenario-based design, parent-child interaction, family, emotion socialisation, emotion regulation, user-centred design, tangible interaction

## Abstract

Parenting practices have a profound effect on children’s well-being and are a core target of several psychological interventions for child mental health. However, there is only limited understanding in HCI so far about how to design socio-technical systems that could support positive shifts in parent-child social practices in situ. This paper focuses on parental socialisation of emotion as an exemplar context in which to explore this question. We present a two-step study, combining theory-driven identification of plausible design directions with co-design workshops with 22 parents of children aged 6-10 years. Our data suggest the potential for technology-enabled systems that aim to facilitate positive changes in parent-child social practices in situ, and highlight a number of plausible design directions to explore in future work.

## 1 Introduction

Parent-child interactions constitute the primary social learning context for children, shaping a number of social-cognitive and social-emotional processes with profound effects on children’s well-being, such as social cognition and emotion regulation [32, 63, 67]. While work in HCI has suggested the potential of digital technology to scaffold parent-child interactions, only limited work has aimed to design digital systems that would intend to change complex parent-child social practices, such as those involved in managing emotions within the everyday parent-child relationship. In such contexts, effecting change involves intervening not only on the level of the individual, but also on the level of the parent-child interaction and the patterns of established dynamics between them – leading to a wealth of socio-technical challenges for digital technology design [114].

In this paper, we explore these design challenges in the context of parents’ role in supporting child emotion regulation development, as a particular example of a key psychological process which is: (i) grounded in parent-child everyday interactions; (ii) core for child’s long-term well being [1, 9, 59, 109]; and (iii) known to be a point of difficulty for many parents [11, 24, 45, 82]. As such, the focus on parental socialisation of emotion [26] allows us to draw on existing in-person parenting programmes [30] for examples of non-technological approaches that aim to affect such dynamics between parents and children, while addressing a so far under-researched design space from an interaction design perspective (cf., [122]). In particular, while the psychologically active *content* of the programmes is well understood and brings clear goals of how parent-child dynamics and established practices could be shifted (e.g., supporting parents to validate their child’s emotional experience and communicate support and acceptance), the existing digital versions of such programmes have only limited approaches to providing *socio-technical support* in-situ: from an interaction design perspective, the programmes fully rely on asynchronous psycho-education components, mostly through web-based modules heavily drawing on reading and lecture-like videos; and lack support for in-situ practice, or even role-play practice, which is common within in-person settings. In summary, the psychology literature provides theoretical and practical grounding for the kinds of parent-child interactions that should be designed for; but digital technology is not—yet—used to to support or scaffold such changes in parent-child daily interactions.

As a first step towards addressing this gap, the present study builds on a novel socially assistive robot—Purrble—that has emerged from prior CSCW and HCI research (cf., [114, 121]) on supporting child emotion regulation. Purrble is a digitally-enabled, child-facing object which can become embedded in family life and offer support to the child in an ongoing way (cf., [57]); while potentially also serving as a catalyst for the delivery of additional scaffolding to influence family social practices around emotion regulation in the context of everyday parent-child interactions. However, it is not yet clear what interaction design mechanisms could provide such support in ways that both (i) utilise intervention components known to be important from psychology; and (ii) fit in with parents’ and children’s needs and interactions.

We are approaching this work as a case study to consider a ‘hybrid’ design approach in this context, with potentially broader applications in other areas of HCI, such as family informatics and technology-supported learning. Specifically, we explore how a *physical* and *experiential* design like Purrble could be enhanced with *digital, cognitive* supports to facilitate parents’ and children’s informal learning in a *situated* manner, within the context of their daily lives. This *family-centred* approach considers the family context and how the system can be used by and affect social practices around emotion regulation for both parents and children, rather than designing only for one or the other.

In this paper, we present a two-step study with the aim to propose a set of design directions for technology-enabled systems aiming to provide parenting support to parents of school age children. In the first step, we drew on prior empirical and theoretical work to identify plausible interaction design approaches for affecting parent-child interactions around emotions over time, and translated them into scenario probes to serve as inputs into a co-design process with parents of young children. In the second step, we involved 22 parents of children aged 6-10 years in co-design workshops to get feedback and new suggestions around the interaction design approaches instantiated in the scenario probes, as a means of developing an understanding of the ways we could design to scaffold complex and shifting dynamics in this particular design space.

On a conceptual level, the paper proposes four theory-driven design directions encapsulated in the scenarios (i.e., prompting parent-child emotion discussions, scaffolding in-the-moment parental support, facilitating experiential learning through storytelling, and enhancing parental attentiveness to child’s emotional experiences), together with a range of interaction design assumptions that could underpin the socio-technical system. Empirically, our findings suggested that parents were in principle enthusiastic about receiving additional parent-child and parent-oriented support, while also identifying a range of specific design challenges to address in future work. Combining these conceptual and empirical contributions, this paper opens up a set of plausible design directions for technology-enabled systems that would attempt to facilitate positive changes in parent-child interaction, with potential broader implications for future research that can be of interest to both HCI and psychology communities.

## 2 Related Work

### 2.1 Technology-Supported Parent-Child Interaction

Much of HCI research on supporting parent-child interaction has focused on *facilitating remote parent-child communication*. Researchers have designed technology-enabled systems to support remote parent-child communication [118, 133], play [37, 53], and joint media engagement (JME)[92]. Other work has focused on *supporting parent-child interactions in co-located contexts*, exploring technology-enhanced storytelling activities [15, 17, 98, 120, 127, 136]; the use of multi-touch interactive tabletop applications to support parent-child interaction [132]; surface haptic technologies to enhance parent-child collaborative science learning [7]; and the impact of interactive elements of tablet-based e-books on dialogic reading [68]. Finally, Yu et al. [135] explored how parents perceive and support their children’s learning with educational technologies at home, arguing for the need to consider parents as both ‘learners’ and ‘scaffolders’ in the interaction and include design features to support both these roles.

A related area of work is family health tracking, with recent work showcasing how parent-child collaboration around tracking can facilitate child’s informal learning during naturally occurring family moments, as well as increase interaction and create bonding moments between parents and children [87, 88, 91, 97]. For example, systems such as Snack Buddy[104], TableChat[75], DreamCatcher[91], and Storywell[97] use features such as child-friendly visualisations, data sharing, and competition among family members to increase family engagement with the system and initiate conversations around healthy living practices (e.g., physical activity, sleep, diet) within the family. Similarly, other work has explored how child-oriented commercial activity trackers can become a tool to have family conversations around healthy habits, support children’s data literacy development, and provide opportunities for parents and children to collaborate towards family health and daily life management [87, 88].

There has also been growing interest within HCI to explore opportunities for technology *to deliver interventions in the home* within the context of everyday parent-child interactions. However, most of these only actively involve the parent. For example, in-situ mobile intervention systems TalkBetter [54] and TalkLIME [115] have been designed to support interactions between parents and children with delayed language development by using meta-linguistic analysis to provide real-time feedback to parents during conversations with their child. SpecialTime provides parents engaged in Parent-Child Interaction Therapy with automatic, real-time feedback on their dialogues with their children, as a means of providing feedback during their at-home practice of the skills taught in therapy sessions [52]. Similarly, TOBY Playpad [80] is a tablet-based tool for children with Autism Spectrum Disorder and their parents, designed to facilitate the delivery of intervention by parents in the home as part of daily routines. ParentGuardian is a mobile application which detects parental stress and provides in-situ cues to parents of children with Attention-Deficit/Hyperactivity Disorder (ADHD) to remind them of behavioural strategies to practice in the moment [90]. HCI researchers have also designed technology-mediated interventions to encourage children to adopt healthy eating habits. For example, MAMAS [58] aims to improve children’s eating behaviour by monitoring their food intake and parent–child interactions around mealtimes, collecting data with self-report questionnaires for parents, and aiding parents to engage in data-assisted self-reflection on the mealtime experiences. Other projects such as MOBERO [116] and WAKEY [14] have explored opportunities to use technology to support parents in establishing routines for their children.

Recently, other HCI work has attempted to impact parent-child interactions by focusing on *supporting parents’ reflection of their role during everyday interactions* with their children. For example, the Awareness Object is a tangible device designed to enhance parent’s awareness of the different roles they can fulfil during a collaborative activity with their child and help them shift their focus to the interaction with their child, by serving as a physical representation of the parent’s possible roles on a continuum ranging from ‘peer’ to ‘mentor’[95]. Other early-stage work has explored how providing parents with a second-person live-view from their child’s perspective during their everyday face-to-face interactions [65], or specifically during moments of conflict [134], could facilitate awareness of how their child sees them in the moment and a more empathic perspective-taking. However, while these systems aim to impact parent-child interactions, they do not involve the child.

Other recent work has suggested the potential of digital technology to *directly support the development of typically developing children’s emotional competencies at home*, by facilitating experiential practice and learning of skills in everyday contexts, and providing the tools to scaffold parents’ engagement with and reinforcement of emotion skills in everyday interactions with their children[112]. However, to best of our knowledge, only a handful of relevant systems have been specifically designed with the aim of involving both parent and child within the interaction. For example, CUPA [72] is a three-component child companion prototype combining heartbeat detection and simulation with deep breathing prompts, and an emotion check-in feature to encourage the child to indicate if they are feeling good or bad; with an ‘emotion review’ feature to give parents’ insight into their child’s emotions over the day, with the assumption that that would enable parents to initiate discussions with their children around their feelings. Wallbaum et al. designed an interactive storytelling prototype aiming to help parents and children explore emotional situations through the recreation of different narratives using a multi-modal interface [127]. Both of these are early-stage projects and have not yet explored the use of the systems within everyday parent-child interactions.

Finally, only two projects have specifically looked at *scaffolding parent-child interactions around emotions*. Slovak et al.’s [113] technology probe explored the potential of technology to enable parent-child interactions needed for social-emotional learning through an interactive, game-like activity, while the ‘smart toy’ project discussed in the introduction and outlined in more detail in section 2.3 ([114, 122] is, to best of our knowledge, the only work which has explicitly targeted parental emotion socialisation practices. Both have provided empirically grounded design suggestions for technologies aiming to facilitate positive shifts in parent-child interactions around emotions in situ: supporting experiential learning; scaffolding the parent’s scaffolding role in the interaction; and designing for the child, the parent, and emergent parent-child interactions.

Overall, while work in HCI has suggested the potential of digital technology to scaffold parent-child interactions, there is currently only limited understanding about how to design socio-technical systems that could support positive shifts in complex parent-child social practices in situ.

### 2.2 Emotion Socialisation & Parenting Interventions

#### 2.2.1 Parental emotion socialisation

Children learn how to understand, regulate, and appropriately express their emotions within close interpersonal relationships and, particularly, through parent-child interaction [63, 67]. Parents engage in emotion socialisation through the way they express emotions, how they react to their children’s emotions, and how they discuss (or not discuss) emotions [26, 27, 29, 83]. Parental emotion socialisation practices comprise behaviours which are either supportive (e.g., discussion of the causes and meaning of emotions, reactions that are emotion-focused, problem-focused, or encouraging of emotional expression) or non-supportive (e.g., avoidance of emotional discussion, minimising or punitive reactions) [26]. These practices have been shown to predict a host of important outcomes, such as children’s and adolescents’ well-being, emotion regulation, behavioural and social functioning, and academic performance [18, 45, 60, 78, 107].

#### 2.2.2 Emotion-focused parenting interventions

Despite the importance of parental emotion socialisation in fostering children’s emotional competence and well-being, only a handful of parenting interventions have specifically focused on helping parents adopt supportive emotion socialisation practices and/or use emotion-focused parenting skills in their interactions with their children [30, 47]. For the last 50 years, research on evidence-based parenting interventions has been based on behaviourist theories and dominated by behavioural management parenting programmes, such as Triple P [103] and the Oregon model of Parent Management Training [38], which typically focus on managing child behaviour, and provide limited explicit teaching of emotional support and coaching skills to improve parental socialisation practices.

The few interventions which explicitly target these practices have typically been adaptations of existing parenting programmes, enhanced with additional components specifically training parents in emotion communication skills, such as Emotion Enhanced Triple P [34, 102], Parent-Child Interaction Therapy-Emotion Development [71, 74], and Parent Management Training for children with oppositional behaviours enhanced with brief training in parent-child emotion communication [101]. However, there is growing recognition of the importance of including emotion communication skills training in parenting interventions, partly due to research showing it is associated to the largest treatment outcome effect sizes [131]. This has led to the development of programmes such as Tuning in to Kids [50], Emotion-Focused Skills Training [2], and Let’s Connect [106], which specifically focus on increasing supportive emotion socialisation in families, by targeting specific emotion-focused parenting practices (e.g., active listening, validating and normalising feelings, emotion awareness and labelling, and problem solving) that can be taught in a skills-based intervention format. The intervention content is usually delivered over several in-person sessions (typically between 6-12 sessions of 2-hour duration on average), relying on mechanisms such as psychoeducation, written exercises, video presentations, role-plays, and home practice [30, 47, 106].

#### 2.2.3 Challenges of existing parenting interventions

In spite of these interventions’ promising effects on parent and child outcomes, such in-person programmes are time- and resource- intensive and struggle with reaching, engaging, and retaining parents [56, 117]. Similarly, although digital versions of traditional, evidence-based parenting programmes are starting to become more common and have attempted to address some of these challenges [19, 124], they typically utilise only a limited set of interaction design mechanisms, such as web-based modules heavily drawing on reading and lecture-like videos, do not provide in-situ support to parents for at-home practice of skills, and still struggle with engaging and retaining parents, with substantial impacts on their efficacy [119, 124].

A common challenge across in-person and digital parenting interventions, then, is the reliance on asynchronous psychoeducation components, and lack of scalable delivery approaches that would provide in-situ support to parents to practice and apply the skills within their everyday interactions with their children. This points to the need for interventions which provide in-situ support for the practice of skills and facilitate experiential learning. Digital technology has the potential to not only offer more constructive in-the-moment support to both parent and child to deal with triggering situations, but also enable them to learn from real-life emotional experiences that are personally relevant to them, rather than relying on role-plays or the recollection of past experiences as is common in parenting interventions. However, it is not yet clear what would be plausible interaction design mechanisms to support this in ways that both (i) make use of evidence-based intervention components ; and (ii) fit in with the parent-child needs and interactions. In the following section, we describe the smart toy intervention that we have selected as a starting point to explore the questions of the present study.

### 2.3 The Smart Toy

The system–smart toy–used in this work has emerged from a 2-year-long co-design process with children aged 6-10 years with the aim to support child emotion regulation practice in everyday settings [57, 114]. The smart toy has since been taken to production by the largest non-profit developer of social-emotional learning programmes in the US, Committee for Children, under the commercial name of ‘Purrble’.

**Fig. 1:**
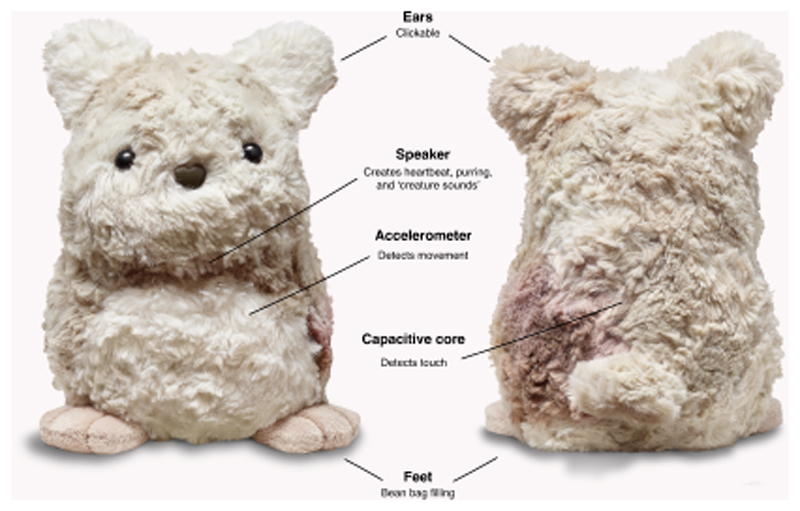
The smart toy

Purrble is a small, interactive plush animal (see Figure 1) presented to children as a ‘vulnerable creature’, which is often anxious (indicated by a fast heartbeat-like vibration), but can calm down when stroked. The smart toy’s underpinning theory of change is unique in relation to existing interventions aimed to support child emotion regulation due to its focus on providing ‘situated support’ and being ‘child-led’: the intervention aims to deliver in-the-moment support (*situated*) that empowers the child to learn how to regulate their emotions without relying on their adults or formal training (*child-led*). The full description of the design process and the intervention theory of change is available elsewhere [57, 114, 121].

The logic model underpinning the intervention suggests that by ‘soothing’ the smart toy, children down-regulate themselves; that children will start seeking the smart toy when they need to calm down or relax; and that repeated engagements will, over time, enable positive shifts in emotion regulation processes in the family, by facilitating the emergence of alternative family approaches and narratives around emotion regulation [114, 121]. However, Purrble currently does not include any additional support resources that would facilitate the latter. The only material for parents currently accompanying the toy is a card stating its purpose (i.e., helping children calm down and strengthen their emotion regulation skills), with simple suggestions on how parents could help their child engage with the toy (e.g., ‘Help your child explore how Purrble is feeling’, ‘Make Purrble part of your child’s bedtime routine’).

This paper builds on recent work on the smart toy [122] which explored if and how it would facilitate additional parent-child interactions around emotions during deployments with 29 families; and involved parents and parenting course facilitators in a co-design process exploring how this could be designed for directly. While the deployment results were promising for a subset of the families, with the toy seemingly facilitating shifts in how parents reacted to and talked about children’s emotions, in the remaining families the toy was predominantly being used by the child for in-the-moment soothing only. The authors noted that additional design work is needed to support parents in adopting supportive emotion socialisation practices through the smart toy intervention and suggested the following two as important design targets: (1) *engaging parents*; and (2) *guiding emergent parent-child interactions*. In the following section, we describe the approach we used to translate these broader design targets into plausible design directions and explore their acceptability with parents of young children.

## 3 Phase 1: Identifying Plausible Design Directions

The aim of the first phase was to explore the key gaps around the smart toy intervention with regards to supporting emotion-related parent-child interactions in the context of families’ daily lives. In particular, we aimed to identify design targets for the smart toy intervention and translate them into probes that would allow our target users to co-produce what a design solution could look like in practice (as part of co-design workshops in Phase 2).

### Design goal

While the smart toy was designed predominantly for ‘situated and child-led’ use, the research team behind its design conjectured that its appropriation in the home could shift family dynamics around emotion in multiple ways (cf., [57]):

(1) through direct in-the-moment calming down support *for the child*;
(2) by introducing a shared *parent-child* narrative to support and enable new social practice to emerge; and, finally,
(3) by providing the *parent* with a set of (cognitive) tools to change their own perspective and approach around their and their child’s emotions.

As mentioned in the previous section, prior empirical work shows that the first aim is well understood, but that additional design support might be needed to enable the other two [122]. In particular, interview data from parents who had had the smart toy in their homes for at least a month, suggested that parents often thought about the toy as: (i) *for their child only* – i.e, perceived by parents as something that is meant to only be used by the child: as children tended to mainly interact with the smart toy when in their room, parents were often unaware of what their child did with it and would tend to forget they even had it; and (ii) *a single-purpose tool* – i.e., something that was meant to be used by the child as a tool to help them calm down or relax when needed and had not thought it could have additional uses.

Some plausible reasons for the misrepresentations of the intended design included a lack of awareness on the part of the parents about the potential benefits of actively fostering children’s emotional competencies; competition with other duties demanding their attention on a daily basis which made it difficult to *“sit and talk about [*…*] emotions”*; and perceived lack of the knowledge and/or confidence needed to scaffold children’s learning around emotional experiences effectively without further support. However, the fact that for some families the smart toy did generate shifts in emotion-related parent-child interactions in all three of the ways outlined above, suggests that there is potential for digital technologies to support such social practice shifts. Therefore, the goal of the first phase of this work was to explore possible design approaches that would emphasise the parent-child, and parent-oriented parts of the system (#2 and #3 above); and then translate them into scenario probes to serve as inputs into a co-design process with parents of young children.

### Design process

To determine viable design strategies for the parent-child and parent-focused aspects of the smart toy system, we utilised three data sources: existing literature in HCI and psychology, as well as data from a previous deployment study of the smart toy involving 29 families with children between the ages of 8 and 10 [122].

To do so, we started by looking at the prior empirical data on the smart toy’s appropriation in families of young children, as well as at psychology literature to explore the key gaps/design opportunities around the intervention with regards to supporting emotion-related parent-child interactions in families’ daily lives. The first author created text descriptions of four scenarios exemplifying some of the most common issues around Purrble’s use as reported in previous published work. These served both as a jumping off point for us to think about plausible design directions, but also to ground the probes (described below) in real-life situations parents might come across when using Purrble without any additional support. The first author then created text descriptions of the proposed interaction design approaches we wanted to explore within each scenario, which were grounded in prior empirical data, and HCI and psychology literature. These were then further refined by the first and second author and got developed into scenario probes, to serve as inputs into a co-design workshop to get feedback from parents and refine future design ideas.

**Fig. 2:**
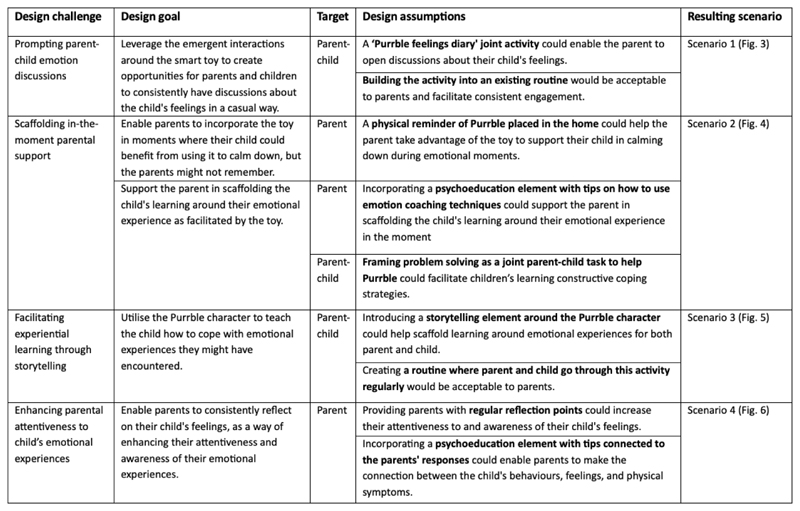
Summary of design challenge, goal, and assumptions for each scenario

In the rest of this section, we describe the four identified gaps/design opportunities in more detail, with a particular focus on the design process and rationale that led to the creation of each respective scenario probe. We hope that the design challenges, goals, and assumptions encapsulated in each scenario probe (see Fig. 2 below for a summary) can be useful more broadly, by serving as a starting point for others to explore similar questions and/or alternative design approaches.

### 3.1 Prompting Parent-Child Emotion Discussions

One of the key design challenges we chose to focus on was how to provide additional scaffolding around the smart toy to enable parents to use it to *open up discussions about the child’s feelings*. This challenge was particularly interesting both due to the toy’s potential to facilitate such discussions as assumed by its logic model and shown in previous studies, as well as due to the importance of parent’s discussion of emotion in the psychology literature. As previously mentioned, the way parents discuss emotions with their children is one of the core emotion socialisation practices and is linked to all key aspects of the child’s emotional competence [28, 43, 123].

#### Design goal

Our main goal was to leverage the emergent interactions around the smart toy in a way that would create opportunities for parents and children to consistently have discussions about the child’s feelings in a casual way (*parent-child oriented aim*). We wanted both to provide parents with prompts to initiate such discussions, as well as allow the child to express their feelings in a non-intimidating way, especially in situations where they might otherwise feel uncomfortable to share their feelings.

#### Design process

Our design approach was inspired by one of the most common ways the smart toy was appropriated by children as reported in previous studies, namely as a proxy to talk about their own emotions to their parents. We decided to explore the idea of a ‘Purrble feelings diary’ the child and parent would fill in together on a smartphone-based Purrble companion app, with prompts on how Purrble’s day was and why Purrble felt that way. The assumption was that asking about Purrble’s feelings instead of directly asking the child, would likely allow the child to bring up potentially uncomfortable feelings in a safe way. We expected that having the parent go through the activity with the child, would give them an insight into the child’s feelings and reasons behind them, allow them to ask more questions, and help the child process and problem solve. To support ongoing engagement and reinforcement of skills, we envisioned the activity could be embedded into an existing routine parents have with their children. Our assumption was that embedding the activity in an existing routine would likely be easier for parents than having to create an entirely new routine, and would enable them to consistently have discussions around emotions until it became a habit. In the scenario, we chose to present this activity as part of the child’s bedtime routine, as this is a time of day when many parents are typically present with their children.

#### Resulting scenario (Fig. 3)

To frame our design scenario for workshop participants, we drew upon an issue reported in prior work on Purrble, where while children would use the toy to calm down or feel better when needed, the toy did not open up any discussions around feelings between parents and children. The scenario was then presented as a potential solution to a situation where the child would come home from school visibly upset, use the Purrble to calm down, but not tell their parent what had happened. The scenario starts by the parent being prompted to start the activity by a reminder on their smartphone. They go through the activity with their child and once the child has expressed the reasons behind their feelings, the parent is shown to be using the emotion coaching techniques of naming and validating emotions, and then comforting the child.

#### Interaction design assumptions

To summarise, this scenario rests on the following assumptions : (1) a ‘Purrble feelings diary’ joint activity could enable the parent to open discussions about their child’s feelings; and (2) building the activity into an existing routine would be acceptable to parents and facilitate consistent engagement.

### 3.2 Scaffolding In-the-Moment Parental Support

Another design challenge was providing the scaffolding needed to facilitate parents’ supportive reactions to the child’s displays of emotion in the moment. Specifically, we wanted to enable the parent to take advantage of Purrble to, first, support their child in calming down, and then once the child had calmed down, help them learn from their emotional experience.

#### Design goal

Our main goal in this context was twofold: First, to enable parents to incorporate the toy in moments where their child could benefit from using it to calm down, but the parents might not remember (*parent-oriented aim*); and, second, to then support the parent in scaffolding the child’s learning around their emotional experience as facilitated by the toy (*parent-child oriented aim*).

#### Design process

Following the two-goal structure, our design work drew on two sets of prior HCI research.

For the first goal, we were inspired by work on ambient and tangible systems showing how physical objects embedded in the user’s environment can act as ongoing ambient reminders (e.g., [86]), and started exploring the idea of an accompanying physical token for the parent. We wanted the object to be associated to Purrble in some way, be visible to parents in moments where the child might need support to calm down, and unlikely to be misplaced.

The research team brainstormed ideas ranging from hi-tech (e.g., a wearable ring or watch for the parent which could detect stress and display a reminder of Purrble) to no-tech solutions (e.g., a photo of Purrble placed among family photos or a ‘nest’ for Purrble). In the end, we settled on the simple solution of a photo of Purrble which the parent would choose where to place in their home. Our rationale was that this still met the criteria we set, while also being easier to eventually deploy and scale than other higher-tech options. We envisioned the parent placing this object in spaces in the home either where conflicts typically arise and/or where they choose to go to calm down before re-engaging with their child. As those spaces are very likely to differ from family to family, we wanted to give parents the choice of where to place it. In the scenario, the photo of Purrble is placed on the fridge in the kitchen where the parent goes to calm down for a moment.

For the second goal, we drew upon emotion-focused parenting interventions which train parents in using emotion coaching/communication techniques to respond in a supportive way to children’s emotions [48, 62, 106, 125]. Guided by the need to provide more explicit skill building to the parent, we envisioned that the Purrble companion app could include psychoeducation modules informing the parent on the basic concepts of emotion socialisation, emotion coaching, emotion regulation, and the associated benefits for them and their children, as well as specific tips on how to apply emotion coaching skills when interacting with their children. By framing problem solving as a task to help Purrble, we drew upon literature on the benefits of pretend play as a medium with unique qualities for enabling the child to gain emotional mastery over situations they might perceive as difficult or scary in a safe environment [40, 81, 93]. We assumed this framing would also facilitate problem solving by putting them in a more empowering position, where rather than being told how they should behave by the parent, they are the ones coming up with solutions to help Purrble in collaboration with the parent. We expected this would also tap into the toy’s persona of a little, anxious creature that needs help to manage its emotions and the associated nurturing relationship the child develops with it (as assumed by the smart toy’s logic model).

#### Resulting scenario (Fig. 4)

The design scenario was framed around the issue of parents forgetting about the smart toy when it is out of sight. The situation we used as a jumping off point was the following: Two siblings are fighting and the parent asks one of them to go to their room to calm down. The child does not respond well as they feel they are being punished and the situation escalates. The parent could have directed the child to Purrble instead, but they did not remember its existence in that moment.

In the scenario, upon the parent seeing the Purrble reminder on the fridge, they open the app to remind themselves of the emotion coaching steps, before going back to the child and attempting to apply them in practice. They name their child’s feelings and prompt them to take some time with Purrble to calm down before they discuss what happened. Once the child has calmed down, the parent goes back to the child, shows empathy, and names and validates their feelings. They then help the child problem solve and come up with more constructive ways of approaching the situation they found hard to manage, by encouraging them to ‘teach’ Purrble how to deal with a similar situation in the future.

#### Interaction design assumptions

In summary, this scenario encapsulates the following assumptions: (1) a physical reminder of Purrble placed in the home could help the parent take advantage of the toy to support their child in calming down during emotional moments; (2) incorporating a psychoeducation element with tips on how to use emotion coaching techniques could support the parent in scaffolding the child’s learning around their emotional experience in the moment; and (3) problem solving would be facilitated by framing it as a joint parent-child task to help Purrble learn constructive coping strategies.

### 3.3 Facilitating Experiential Learning Through Storytelling

The previous scenario engages with the idea of supporting the parent in facilitating the child’s learning after an emotional experience. However, the proposed ‘solution’ assumes that the parent would be able to apply the emotion coaching skills in the moment and guide the child through this process without any additional scaffolding. This led us to start exploring plausible design directions to directly scaffold the learning process for both parent and child outside of the emotionally active moments (cf., [111]) and enhance the parent’s ability to co-construct emotionally supportive narratives about the child’s emotional experiences, which has been shown to be critical for the child’s emerging cognitive and social-emotional development [34, 35, 105].

#### Design goal

The main goal was to explore the potential of using the Purrble character to teach the child how to cope with emotional experiences they might have encountered (*parent-child oriented aim*). We aimed to envision approaches to support parents in drawing on narrative and storytelling to craft learning experiences for the child: in particular we were interested in interaction design approaches that would make it easy for parents to share new ‘knowledge / tips’ with their children, as well as have the opportunity to reflect on previous difficult situations with the child, once the emotions have died down, drawing on the potential of using Purrble as a third-party catalyst to ease such discussions (cf., [20] for discussions on similar approaches to using digital technology as the ‘third partner’ in mental health interventions).

#### Design process

We drew on the power of narrative and stories to engage young children with emotional content. Interactive technologies such as tangible storytelling systems ([13, 127] have been demonstrated to bring parents and children into a collaborative relationship with one another [55, 113], especially within the ‘magic circle’ of play [100] (see [46] for a review of the rich body of work within HCI exploring the design and use of storytelling systems for parent-child interaction). Building on the idea of problem solving as a joint parent-child task to ‘teach Purrble’ constructive ways to manage its emotions, we envisioned introducing a storytelling element, whereby the parent and the child would jointly read stories the Purrble character goes through, linked to emotional experiences the child might come or might have come across. The stories are leveraging Purrble to help the child reflect on emotions and behaviours, and come up with constructive ways of managing their emotions outside of heated moments. We chose to frame this as a joint parent-child activity rather than having the child go through it on their own as a means of promoting parental involvement, ensuring the parent could support the child further if needed, and as a way of crafting a shared set of narratives that both parents and children could refer to later. To scaffold this, we envisioned incorporating questions to help the parent facilitate the learning for their child, directly drawing on emotion coaching and similar approaches taught to parents in emotion-focused parenting programmes, such as labelling emotions, and helping the child problem-solve. For example, a list of feeling words that could be applicable in the situation is displayed on the respective part of the activity: enabling the child to choose the appropriate feeling word from the list (while also expanding their emotion literacy [23, 34, 101]), while the parent could provide explanations for their meanings if needed.

#### Resulting scenario (Fig. 5)

The scenario was framed as a potential solution to the issue of the smart toy’s impact on the child’s emotion regulation not extending to situations when it is not available to the child. In the scenario, the parent is first prompted to select a challenging behaviour that they would like to help their child work on through the activity. Then are then presented with a story where the Purrble character exhibits a similar behaviour. In the activity, the child is prompted to select how Purrble might be feeling in that situation from a range of feeling words. They are then prompted to reflect on how they would feel in a similar situation, ‘teach’ the Purrble good coping strategies to use in such situations, and finally reflect on how they themselves could use them to apply them in similar situations they might encounter. The parent is doing this activity with the child to help guide them through these steps and make sure the coping strategies the child comes up with are appropriate.

The activity is designed to help the child first take Purrble’s perspective and then remember how they felt in similar situations. For example, after the story is presented, the following emotion-coaching inspired questions appears : “How do you think Purrble might be feeling in this situation?". This is then followed by “Have you ever been in a similar situation? Share how you felt with Purrble!”. We expected that engaging with this activity could nurture the child’s empathy and understanding of other’s emotions by helping them practice taking others’ perspectives and identifying their feelings, while at the same time strengthening their understanding of their own emotions.

The last steps of the activity are then about problem solving. Here, we wanted to build in more scaffolding to support the parent and child in problem solving. The child is first prompted to come up with good coping strategies for Purrble (e.g., “How can Purrble stay calm when things don’t go his way?”), before reflecting on what strategies they could themselves use in a similar situation (e.g., “What can *you* do to stay calm in a similar situation?”). We expected this would help parent and child to draw connections between what is happening within the ‘Purrble stories’ to real situations in the child’s life. Finally, to promote repeated engagement and practice crucial for the reinforcement of emotional skills, we envisioned this as a routine activity the parent would do with the child at regular intervals (weekly in the scenario).

#### Interaction design assumptions

In summary, this scenario encapsulates the following assumptions: (1) introducing a storytelling element around the Purrble character could be a plausible way of scaffolding learning around emotional experiences for both parent and child; and (2) creating a routine where parent and child go through this activity regularly would be acceptable to parents.

### 3.4 Enhancing Parental Attentiveness to the Child’s Emotional Experiences

The final design challenge was increasing parents’ attentiveness to and awareness of their children’s feelings and behaviours. Parents’ ability to effectively attune to their children’s emotional experiences has been seen as being intrinsically connected to their ability to use supportive emotion communication skills in their interactions [64, 106], and was, thus, selected as an important design direction to explore.

#### Design goal

The main goal here was to enable parents to consistently reflect on their child’s feelings, as a way of enhancing their attentiveness and awareness of their emotional experiences, an important first step to then responding to them supportively (*parent-oriented aim*).

#### Design process

In attempting to address this problem, we took inspiration from prior work on Purrble reporting that the daily surveys parents had to complete on their child’s feelings and behaviours as part of the study made them more attentive to their child’s emotions and reactions to emotionally evocative situations. This finding is also in line with literature on measurement reactivity [69, 77], self-monitoring [42], and question-behaviour effect [128], showing that people’s behaviour, emotions or cognitions can change due to being measured as part of a research study. As a result, we chose to envision a similar mechanism where the parent would receive a reminder to complete a few questions about their child’s day and feelings at regular intervals — our main interest in this very low-tech approach was to see the reactions of parents to such interaction design in general.

#### Resulting scenario (Fig. 6)

In the scenario, the parent receives a reminder to complete a short daily survey about their child’s day and their feelings through a smartphone-based app. We purposely chose the frequency to be daily, both because that frequency was found to be helpful for parents in prior work on Purrble [122], but also because we assumed parents would potentially find it extreme outside the context of a study, which would likely generate interesting feedback in terms of what would be acceptable and feasible in their daily lives. We decided to frame the resulting scenario around a situation where the child is experiencing a physical symptom that neither the child nor the parent realise is due to stress, as a means of demonstrating to participants how being attentive to their children’s behaviours and feelings could be of benefit of them in a real-life situation which is common for many young children. We also incorporated a psychoeducation element in the form of a tip connected to the parent’s responses to the questions. In the scenario, the tip is that physical symptoms which appear before potentially stressful moments might be a sign that the child is experiencing stress. This is then followed by a suggestion to help their child name their feelings and make the connection between their stress and their physical symptoms. In the scenario, parents refer to Purrble as an example of how feelings might manifest in the body (e.g., “Like your Purrble, we often feel our emotions in our bodies. So, sometimes our tummy might hurt when we are feeling anxious").

#### Interaction design assumptions

To summarise, the assumptions underpinning this scenario are the following: (1) providing parents with regular reflection points could increase their attentiveness to and awareness of their child’s feelings; and (2) incorporating a psychoeducation element with tips connected to the parents’ responses could enable parents to make the connection between the child’s behaviours, feelings, and physical symptoms.

### 3.5 Summary of Proposed Scenarios

To summarise, the four scenarios above were designed to targeting a number of distinct plausible design directions that would support parent and child emotional functioning. Our particular focus was on drawing on HCI and psychology literature to identify possible interaction design assumptions (e.g., use of physical reminders, scaffolded parent-child narratives, or ongoing reminders) that could lead to parent-child experiences that are well theoretically grounded in psychology literature, all while boiling these down into highly simple scenario forms that could be presented to parents—who have lived experiences with having Purrble in their homes—in co-design workshops to elicit their perspectives, and serve as baseline for co-producing further iterations of design directions seen as potentially helpful. The next section turns to this first empirical test of the proposed design ideas, and their instantiations into specific scenarios.

## 4 PHASE 2: CO-DESIGN

In the second phase, we involved 22 parents of children aged 6-10 years (who had had access to smart toy units for at least a month, and thus were already able to observe any potential change in their parent-child interactions) in co-design workshops to get feedback and new suggestions around the interaction design approaches instantiated in the scenario probes, as a means of developing an understanding of the ways we could design to scaffold complex and shifting dynamics in this particular design space.

We note that the current work has primarily focused on understanding parents’ perspectives on the design approaches used in the scenario probes. As discussed in section 3, previous empirical research suggests that while the smart toy alone seems to be sufficient for achieving the intervention’s child-oriented goal of providing direct calming support to the child, additional support is required to engage parents with the intervention and achieve the parent-child and parent-oriented objectives [114, 121, 122]. Therefore, we focused on understanding parents’ perspectives on what they would be willing to engage with and what we could design to better support their role in fostering their child’s emotion regulation development. However, as children may have unique views, needs, and goals in regards to the use of such an intervention, which may differ from those of their parents, it is crucial that future work aims to understand children’s perspectives and involves them in co-design efforts to explore what they need and are willing to engage with.

In what follows, we first outline the methods, and then move to the qualitative findings, where the parents’ reactions are presented scenario by scenario, with specific focus on parents’ reactions and suggestions relevant to each of the underlying interaction design assumptions. Finally, section 4.3 steps back to synthesise insights across all four scenarios.

### 4.1 Methods

#### 4.1.1 Procedures

Participants were recruited through Mumsnet, one of the most popular parenting fora in the United Kingdom, and snowballing. The study ad included a link to a Qualtrics form where participants could read the study information sheet and complete the study consent form if they were interested in taking part. We deployed the smart toy with 33 parents of children aged 6-10 years for 4-6 weeks, 22 of which were then involved in the co-design process. The relevant ethics approvals were obtained and all participants signed consent forms prior to any data collection.

#### 4.1.2 Participants

All co-design session participants were mothers who resided in the United Kingdom. Most were married (86.4%) or living together with the other parent (9.1%), and had more than one child (90.9%). The majority of the participants were White British (95.5%) and had obtained a bachelor’s degree or higher qualification (90.9%).

#### 4.1.3 Co-design sessions

We conducted 10 co-design sessions with 22 parents in total. The main co-design activities involved reacting to the scenarios and providing feedback and new suggestions, as a means of understanding the acceptability of the design directions they instantiated and uncovering any additional user needs. The co-design sessions took place between 4 and 6 weeks post-deployment, lasted two hours and were conducted over video chat and Miro, an online collaborative whiteboard platform.

Due to scheduling issues, it was not possible to schedule group workshop sessions for the last 6 participants. We decided to have those participants go through the co-design activities individually while on video call with the first author. Each activity on the Miro board was set up so that the participant would first respond on their own and then the first author would reveal a selection of other parents’ responses, to which the participant could then react and discuss with the researcher. We decided on this process to enable the participant to express their own ideas without being influenced by previous participants’ responses, but then still give them the opportunity to reflect and comment on what others had shared.

#### 4.1.4 Data analysis

The audio recordings of the co-design workshop sessions were automatically transcribed in Microsoft Stream. The transcripts were read and checked for accuracy by the first author. The data from these transcripts were analysed following Braun and Clarke’s Thematic Analysis framework [10]. Most of participants’ responses to the workshop activities had been captured on sticky notes on Miro during the workshop sessions. The first author then reread the transcripts, made brief notes on the key quotes and ideas in each workshop session, and added more sticky notes on Miro with any relevant participant responses that had not already been captured during the workshops. Those notes were then coded and grouped together to identify patterns and generate themes across workshop sessions and participants in an iterative process. This approach enabled us to visualise and draw linkages between the codes, which were subsequently clustered into themes.

### 4.2 Findings

In this section we discuss the key findings for each of the design scenarios (see Fig. 2 for a brief summary of the design challenge, goal, and assumptions for each scenario). To protect anonymity, participants are referred to by using ‘P’ for participant, followed by a participant number. Paraphrasing is indicated by words surrounded by brackets.

#### 4.2.1 Scenario 1 – Prompting parent-child emotion discussions

The first assumption explored in this scenario was that a ‘Purrble feelings diary’ joint activity could enable the parent to open up discussions about their child’s feelings. The response to the premise of the activity was overall positive, with parents recognising its potential to help them initiate such discussions in a non-intimidating way, and to, over time, turn it into a natural practice without the need to use Purrble.

“[It’s a] good routine to do daily and bond, have that special connection with your child. Over time, the child would likely get more used to the parent asking questions about those things and might not need Purrble to do that.” [P18]

However, most participants were unsure whether their child would actually express their own feelings through Purrble and expected they would more likely *“make up a story about how Purrble feels rather than speak about their own feelings through Purrble” [P6]*. Parents thought this would be especially true for children who have not developed a bond with Purrble, or for those who are more literal in their thinking and see Purrble as an inanimate object incapable of having feelings. Interestingly, several parents thought that doing this activity would make them feel as if they were trying to trick their child, and/or that their child would *“see through this and not open up” [P15]*. Some of the alternatives proposed were framing the activity as a joint diary or chat between the child and Purrble to allow for a more natural exchange rather than relying on the child talking through Purrble, and having additional prompts to allow the child to talk about their own feelings more directly. Another suggestion was to have the child complete the activity on their own, with the option of sharing their responses with the parent. The general consensus was that parents should be given the option to customise the framing of the activity according to what works best for the child.

Parents’ response to the digital nature of the activity was mixed. Although parents thought that filling out the diary on a phone or tablet would likely be appealing to their child, most where not keen on introducing more technology to engage the child. Common concerns were a that it would be *“too distracting"* for the child, as well as that using screens before bedtime *“would cause more problems than help” [P3]*.

The second assumption this scenario rests on is that building the activity into an existing routine would be acceptable to parents and facilitate consistent engagement. Overall, while participants seemed to appreciate the value of the activity in helping them *“get into the habit of talking about feelings more and having regular slots to do that” [P20]*, most parents appeared to feel strongly against the suggested daily frequency of the activity. Most of the parents agreed that receiving a daily reminder to do the activity would be *“too much"*, feel *“intrusive"*, and make the activity feel *“like yet another thing [they] have to do"*. However, participants recognised that a less frequent prompt would be useful to remind them *“to make it part of the routine” [P2]*, at least until it became a habit.

“[I’m] hesitant about the daily frequency, it might be a bit much. But if you did this regularly, the child would probably start talking more about it without the parent prompting them. [It] could become a natural part of the routine.” [P1]

The specific timing of the routine suggested in the scenario was also seen as potentially problematic by several parents, mostly due to the concern around using screens before bedtime. Despite these concerns, participants were willing to try the activity and embed it into a routine if they saw substantial benefits.

“I don’t love screens at bedtime, but I would do it if I felt the benefits outweighed how much I don’t like it [and] made it something worth using.” [P15]

Overall, the consensus was that parents should be able to customise the frequency of the notifications and the timing of the routine to fit their preferences and needs.

#### 4.2.2 Scenario 2 – Scaffolding in-the-moment parental support

With regards to the assumption that placing a physical reminder of Purrble in the home could help parents take advantage of the toy to support their child in calming down during emotional moments, responses were, again, mixed. Parents seemed to relate to the issue of forgetting about Purrble *in the heat of the moment* and several participants thought the reminder would be *“useful to break the cycle and pattern of behaviour that’s so hard to break in a heated situation” [P01]*, at least until they *built the habit* of directing the child to Purrble in such moments. However, others doubted the usefulness of a physical reminder, as they thought it would be unlikely for them to notice it in moments of heightened emotion.

“In the heat of the moment I don’t know what my response would be.. Even if I walked in the kitchen, would I notice the photo on the fridge?” [P11]

Participants talked about how difficult it can be for the parent to take time to pause and think and/or calm down before reacting when in the midst of such situations, and many stressed this needed to be the first step in the process before engaging with the child.

“[I would add] a small prompt to remember to pause and breathe. For me it’s so easy to react and make the situation worse! Then ask your child to huggle (sic) Purrble and then gently ask about how she’s feeling once calmer.” [P19]

Interestingly, several participants were unsure whether their child would react well to being directed to Purrble in such situations, as they thought it would feel punishing to the child. This finding is in contrast with that in [122], where participants reported that directing the child to Purrble in those moments was perceived by children as something useful they could do to calm down rather than something punitive.

The assumption that incorporating a psychoeducation element with emotion coaching techniques could support the parent in scaffolding the children’s learning around their emotional experience appeared to be supported by parents’ responses to this scenario. Participants thought the reminder of the emotion coaching steps could be a useful support for parents when they are in the midst of a heated situation.

“I think this would work. It can be too easy to get lost in emotion and having a reminder of coping strategies gives the parent a second to calm themselves first and then help their child calm down without the situation escalating.” [P10]

However, a few participants noted that the scenario still relied on the parents’ capacity to take the time to notice the Purrble reminder and then remind themselves of the emotion coaching steps on the app, which they thought was unrealistic to expect of parents in such moments.

“I do not think I would open a Purrble app in a heated situation. The solution to diffusing a heated situation needs to become second nature to really work.” [P9]

It was indicated that some additional prompt would likely be needed until parents had learned to initiate these behaviours habitually. Some of the workarounds suggested were the following: a visual reminder of the five steps of emotion coaching instead of or in addition to the Purrble reminder; allowing the parent to set notifications preemptively for situations when they would expect things to come up; and a Purrble reminder on a smartwatch, which would be prompted by the parent’s raised pulse.

“My watch notifies me to breathe / walk if heart rate increases. I’d personally prefer a Purrble emoji. That would probably calm me down and act as a reminder.” [P11]

In line with our assumption when developing the scenario, participants responded positively to the framing of problem-solving as teaching Purrble more appropriate reactions to a similar situation. Participants talked about how this could be a *“very powerful way of helping the child think how they could react differently to situations and speak out their intentions for the [following] time” [P1]* in a *“collaborative and non-shaming” [P07]* manner. As participant 20 put it, *“I’m not giving [them] a lecture. We’re coming up with ways to deal with these situations together."*. Finally, as we saw in the previous scenario, a few parents thought that their child would find it difficult to engage with the idea of teaching Purrble due to being more literal in their thinking, and suggested a more direct problem-solving approach might work best for them.

#### 4.2.3 Scenario 3 – Facilitating experiential learning through storytelling

The first assumption underpinning this scenario was that a storytelling element around the Purrble character could be a plausible way of scaffolding learning around emotional experiences for both parent and child. This was the most well-received idea in the workshops, with parents recognising several potential benefits of this activity, in alignment with the design goals which guided the development of this scenario. In particular, parents talked about how the storytelling element could prompt discussions about different kinds of situations and emotions the child might have struggled with in a way that felt safe for the child to explore, due to the focus of the story being on Purrble rather than on them.

“I like the educational aspect of working through a scenario to learn about emotions and behaviour. I find my child is much better at analysing the behaviour of other children as it takes the heat out of focusing purely on themselves.” [P14]

Parents particularly liked how the scaffolding built into the activity guides both parent and child through the process of talking about and *“reflecting on emotions not at the immediate moment of the difficult emotion” [P3]*, while also giving the child the language to *“verbalise their emotions and understand what happened” [P2]*.

“I like the way it walks you through the process because it provides some structure. And if you don’t know what you’re doing… Because they don’t come with a handbook. Then you need that.” [P22]

Some participants thought more scaffolding might be needed for the problem solving part of the activity, such as examples of good coping strategies for each particular situation displayed on the screen as potential choices, and another screen showing the positive consequence of better behaviour as a way of reinforcing the outcome of the desired behaviour. Another point raised by participants was that the wording would likely need to be adjusted depending on the child’s age or developmental stage.

Overall, participants liked that the activity was screen-based, as they thought it would be more engaging for children, while also allowing them to go through it *“anytime and anywhere [since they] would never go anywhere without [their] phone” [P22]*. However, some expressed reservations as to the appeal the activity would hold for their child after going through it a few times and suggested that making it more interactive by, for example, gamifying it, might make it more engaging.

With regards to the design assumption that parents would be willing to go through this activity consistently, most parents’ reaction was positive, as they saw benefit in having a joint routine which would allow them to discuss and reflect on situations their child struggled with at a calmer time. Nevertheless, parents’ had different preferences with regards to the frequency of the routine. Some preferred an ‘as and when’ approach, whereby they would go through this activity only when something would happen and they would need their child to work through it. Others envisioned the activity also as a tool to use in advance of particular situations they expected the child might find stressful or upsetting, such as to discuss worries about upcoming events, and thought that setting some time aside weekly to go through the activity would be a good way of dealing with such situations proactively.

#### 4.2.4 Scenario 4 – Enhancing parental attentiveness to child’s emotional experiences

Participants’ response to having daily reflection points about their child’s behaviours and emotions was mixed. Overall, participants thought that being prompted to think about what has happened during the day and what impact that could have had on the child would be useful. However, several parents did not see value in doing this daily and would rather be able to set their own frequency or do it only when they had concerns. Beyond the proposed frequency being too often, some participants felt strongly that this level of support was excessive and unnecessary, *“taking away from intuitive parenting” [P12]*.

“I think I can generally notice how [my child] is feeling and any trends in behaviour, I don’t need an app. It feels quite patronising to parents. It’s not something I need help with.” [P3]

However, it was recognised that other parents might require this level of support in order to identify triggers for specific behaviours. Participant 14 talked about how having this would have been useful when her child was displaying physical symptoms, which turned out to be stress-related.

“This is a really good idea. We had a similar situation a year or so ago when my daughter displayed symptoms of a UTI, but when this was ruled out we were asked to get her tested for type 2 diabetes which was a super worrying time. In the end it was stress/anxiety causing frequent urination and extreme thirst. It’s so easy to assume there’s a physical illness with children.” [P14]

Interestingly, several participants took this a bit further, talking about how it would be useful to add a visualisation element, as a means of helping the parent identify patterns they would have not been aware of otherwise.

Participants talked positively about the psychoeducation element as they recognised the importance of making the link between emotions and physical symptoms, both for the parent and the child. However, a few parents thought the daily reflection points were not necessary and that just having this information would be enough to educate or remind the parent of this link. Finally, several participants pointed out that it might be hard for some parents to help the child draw connections between physical symptoms and emotions and suggested the system could provide more scaffolding to this end, for example in the form of prompts with specific phrases the parent could use to address the situation.

### 4.3 Summary of the Findings

The aim of the scenario probes was to explore together with parents a set of possible socio-technical design directions, with the goal of impacting parent-child social practices around emotions in the context of families’ daily lives. Our specific focus was then on targeting the parent-child and parent-oriented parts of the process, drawing on the child-oriented effects of Purrble as a catalyst. In what follows, we synthesise participants’ responses across scenarios to summarise what we have learned about the reactions to the proposed interaction design assumptions, as instantiated in the presented scenarios.

Overall, parents responded positively to the interaction design approaches instantiated by each scenario. Storytelling seemed to be the most well received, which is a promising outcome from an interaction design perspective given the range of potential design choices around the level and kind of scaffolding built into it, as well as pre-existing work on joint media engagement in other contexts (cf., 5.1 in Discussion). Parents were also interested in using techniques drawn from parenting intervention literature, although some felt they would likely need more scaffolding to be able to apply these techniques in practice with their children.

Embedding the suggested activities into pre-existing routines appeared to be a plausible way of helping parents build habits around them. However, it was clear from the workshops that the burden to the parent needs to be as minimal as possible and well-balanced with the benefit they receive from the activity. Parents seemed to otherwise be tempted to have some of the joint parent-child activities as child-facing only, which would substantially limit any of the intended effects on parent-child social practices around emotions. For example, most participants were resistant to the high frequency suggested for some of the activities. This opens an interesting design question around the ‘dose’ of the activity/intervention needed, both to affect the psychological targets (cf., work on digital health interventions [33, 76, 79] and Ecological Momentary Interventions in psychology [89]), as well as lead to habit formation.

Another design challenge emerging from our co-design data is around the need to provide sufficient scaffolding to build routines while ensuring that the burden for the parent remains minimal. While parents were willing to try things out to see what works, they also then wanted to have the ability to customise the suggested activities depending on what works best for their family (e.g., narrative framing of the activity, routine frequency). In particular, a crucial observation was that an ‘acceptable’ level of scaffolding seemed to be dependent on parents’—and their children’s—perceived needs: what might be perceived as helpful by one parent, could easily feel patronising to another parent who feels they do not need that level of support. This points to the need to build flexibility into the design and give parents choice to select their own goals and ‘solutions’; and the importance of incorporating parents’ perceived self-efficacy and child’s specific difficulties into the design process. From a psychological intervention perspective, an open design question is then how to strike the balance between the inclusion of ‘mandatory’ activities considered key to effect change in terms of psychological targets; and allowing parents to customise and prioritise activities according to their needs and situations, so that they are motivated to keep engaging with the system.

With regards to the proposed approaches to help parents initiate routines, the response was mixed. Several participants expressed resistance to receiving reminders and uncertainty whether they would actually notice cues placed in the home. While there are only limited conclusions we can draw without trialling these in in-the-wild deployments with parents, this is likely an area where further design will be required (e.g., attempting to incorporate sensing or other automated systems, as suggested by some participants). In contrast, the findings also show the parents’ need—and interest—in receiving in-the-moment support with regulating their own emotions first before they can help the child in the ways suggested. This is aligned with and increasingly being recognised in parenting literature [45], with emotion-focused parenting programmes adding components specifically focusing on parental emotion regulation as an intervention goal (e.g., [49, 106]).

## 5 Discussion

We started this paper by highlighting the very limited body of literature that would examine designing socio-technical systems that could positively affect complex parent-child social practices, such as those around parental socialisation of emotion. We also emphasised how such systems could shift these family practices by affecting three different aspects: by providing child-specific support (as the smart toy does); by scaffolding new types of parent-child interaction; and/or by facilitating a change in how the parent might approach or think about these interactions.

Our conceptual contribution so far was to identify four plausible design directions—by drawing on both theory, HCI work, and prior empirical findings—and instantiate these into simple scenarios that then allowed us to use these as co-design prompts with target parents (Section 3). The empirical contribution then explored the acceptability of these ideas, through a co-design process with 22 parents who had had access to smart toy units for a month, and thus were already able to observe any potential changes in their interactions with theire children.

In what follows, we first step away from the specific of the scenarios to discuss how similar work can draw on—and feedback to—other HCI areas, including digital storytelling, joint media engagement, personal and family informatics, and behaviour change in families. We then look towards the potential implications of this work outside of HCI, with a particular focus on parenting interventions.

### 5.1 Designing to Support Parent-Child Interaction

As outlined in the findings, the parents reacted positively to receiving additional scaffolding for all aspects of the three-part model (child-, parent-, and parent-child support), although the responses to the specifics of the proposed varied scenario to scenario: several of the underlying interaction design approaches received particularly strong support (such as storytelling), while others (such as routine development) were seen as practically more challenging.

#### 5.1.1 Storytelling as exemplar design direction

From an interaction design perspective, the positive reception of storytelling elements to scaffold learning around emotional experiences both for parents themselves and their child suggests an exciting connection to other areas of work: On one hand, it is in alignment with prior work on the power of narrative and stories to engage parents and children, both in terms of creating pleasant shared moments; and to create a common narrative to make sense of difficult experiences (cf., [113]). On the other, these insights can connect to the wealth of HCI work on shared parent-child storytelling experiences in other contexts, as opportunities to promote playful and elaborated interaction [108]; as well as with work incorporating playful and game-like components in the interaction between parents and children has been found to be beneficial in motivating parents and children to continue using the technology [46, 68]. Recent research exploring the role digital storytelling can play in the family, suggested that for parents to engage with digital storytelling, these experiences should be designed to provide them with control over the type of story they want to create, and to flexibly support them to achieve their unique storytelling goals, which are influenced by various factors such as the child’s needs, family culture, and the parent’s values [126].

Similarly, prior work on parent-child joint media engagement shows the benefits of parents and children engaging in discussions and meaning-making together when using media, such as enhancing children’s learning from digital content and understanding how it relates to family values [22, 31, 94] – suggesting a broad knowledge base to guide any future development in the context of emotion socialisation too. Importantly, prior work also highlights the socio-technical impacts of design features such as one-sided interfaces, paradigms demanding continuous attention, and lack of support for parallel parent-child interaction, which have been shown to preclude joint participation and play [51]. It is, therefore, important for future design work to take into account how to provide natural opportunities for simultaneous mutual engagement for both parent and child, to prevent app experiences from ending up solitary ones.

In a similar vein, work in family informatics argues for supporting family-centred instead of siloed tracking as children mature and become capable of engaging in such activities, and showcases how parent-child collaboration around tracking can not only promote child’s informal learning during naturally occurring moments, but can also increase interaction and foster bonding moments between parents and children [87, 88, 91, 97]. Together, these insights suggest that storytelling can not only be an enjoyable shared activity between parents and children, but also a powerful way to reflect on personally meaningful experiences and positively impact family practices and informal learning around emotions—especially when combined with evidence-based psychological content.

#### 5.1.2 Challenges with routines and habit building

In contrast, the approaches that psychological interventions might see as core in supporting parenting change, such as reflection points or routines, seemed to be the most difficult ones to design for well. While the participants reflected positively on the goals and potential benefits of the proposed activities, the data suggests a strong push-back against some of the proposed approaches to scaffolding habit building, with scenario components including repeated prompts to initiate routines or behaviours seemingly being particularly problematic.

Our data indicates that while parents overall recognised the potential benefit of the proposed activities (i.e., promoting child’s emotional competence, reducing behavioural difficulties, etc.), they were less motivated to make the effort to engage routinely in such activities. This appeared to be partly due to the promise of reward being too far into the future, not tangible, as it is unlikely that changes in their child’s behaviour or emotion regulation skills would be immediate. Changing one’s behaviour to adopt better parenting practices requires substantial effort, as applying newly learned skills and breaking old habits is notoriously challenging [41, 84, 85, 130], particularly during moments of heightened emotions, such as when a child is having a tantrum [21]. In what follows, we outline three potential design directions to support parents in building new habits.

##### Supporting parents’ reflection on their goals and motivations

Supporting parents’ reflection on their goals and motivations, as well as what elements work best for them and their children is another important design goal for future work. Our observations suggest the need to design routine and habit formation components in ways that would—ideally—provide an immediate benefit to parents, in a similar way to the benefit Purrble provides the child. Recent work on digital health interventions has proposed an effort-optimised intervention model, where the design of the intervention is directly related to increasing the chances that the user will engage and reach their goals despite competing demands [4]. Incorporating components of this model would mean, for example, helping parents find meaning in choosing to engage in the proposed intervention activity rather than in something else, as well as recognising parents’ effort and reframing it as an investment towards the process and their goals. This could potentially be achieved by documenting and rewarding effort over outcomes, and implementing feedback that highlights the aspects that are most meaningful to the particular individual, such as spending quality time with their child, investing time and effort in building a good relationship with them, or improving their child’s skills. We expect that incorporating elements of reflective design [5, 6, 16, 36, 111] might support parents in prioritising across the many possible actions according to their needs and context, and decrease any feelings of being overwhelmed when presented with too many choices. One potential design direction would be introducing gaming elements (for both parent and child) incorporating behaviour change techniques, as common in health behaviour change( e.g., [25]); as well as drawing on such techniques to identify the level of engagement that is needed for habit formation and psychological change (cf., dose-response effects mentioned in section 4.3).

Another related area of work is that on self-experimentation systems [61, 70], which have shown promising effects for individual health behaviour change, although it is unclear if and how this approach could be translated to a more complex design space, such as parenting. Similarly, another potential path would lie in embedding intervention customisability and specifically designing for an iterative process of experimenting with alternative approaches [66].

##### Utilising social modelling to validate caregivers’ efforts and motivate them to engage

We argue that a key consideration when designing to impact family practices in this sensitive and value-laden space, should be about carefully working with parents’ feelings and perceptions of their parenting ability and experiences. Implementing features that facilitate social modelling could help create a sense of community and connectedness among parents who experience similar circumstances and are working towards similar goals. Prior work on fitness tracking tools for families has highlighted the potential of technology-supported social modelling for enhancing parents’ self-efficacy and outcome expectations around family physical activity, two key attitudes for inducing healthy behaviour [96, 97]. For example, Saksono and colleagues’ StoryMap system [97] facilitated the exchange of fitness stories and data among families, enabling them to learn from other caregivers’ approaches in being active, both in terms of how to achieve the desired behaviour, as well as the positive outcomes of the behaviour. Hearing other families’ stories around physical activity validated caregivers’ efforts in supporting their children’s well-being and reduced the feeling of isolation they often experienced as caregivers.

Social modelling could also be a potentially useful approach to validate parents’ parenting experiences and efforts to engage with the intervention. For example, this could be designed for by receiving motivating cues in the form of other parents’ ‘success’ stories when engaging with intervention activities, highlighting other parents’ challenges (e.g., difficulties with engaging or achieving one of their short-term goals due to competing demands or circumstances beyond their control), as well as potentially enabling them to share plans for or past experiences of overcoming these hurdles. Such an approach of sharing similar experiences could potentially be more motivating in this particular context, rather than comparing one’s efforts and performance to that of other parents, which could negatively impact parents’ self-efficacy [129].

##### Embedding intervention activities into existing routines

Finally, a simpler but interesting insight with potentially broader applications is most of our participants’ resistance to the idea of receiving reminders. Instead, they preferred the intervention content built into everyday, joint parent-child activities, which could be embedded into pre-existing routines. Specifically, as discussed in section 5.1.1, interactive storytelling was highly favoured by parents as a method of prompting and scaffolding discussions around emotions with their children, especially around experiences that were personally meaningful.

### 5.2 Implications and Opportunities for Parenting Interventions

While most of the work above is deeply rooted in psychological knowledge about active components of change, the research could also feed back design ideas to the psychology domain. Specifically, a known key issue with existing programmes is in the lack of techniques for transfer and situated support for families and children (cf., [110, 113]). This is also what makes the smart toy approach to be so far unique as a first in-the-moment, digitally supported tool for child emotion regulation [57].

The work presented in this paper attempts to take such novel intervention delivery mechanism one step further: by providing explicit, cognitive-based supports to impact parent-child dynamics. In particular, this would suggest the potential of Purrble—or other digitally mediated in-situ interventions—to be combined with supports to be embedded in families’ homes and used as an adjunct to treatment modalities aiming to effect changes on common domains of emotion-focused parenting (such as parent emotion competence, conflict-resolution skills, or parental empathy and associated constructs). Although the specifics of the design scenarios above were aimed at impacting parent child interactions around emotion, the underlying interaction design assumptions could have broader use and implications for parenting interventions.

Similarly to other HCI work on designing to impact family practices, we argue for a more family-centred design approach in designing parenting interventions, which considers the family as an interconnected system (e.g., see work in family informatics [87, 91]), and allows for situated learning and application of skills. Traditional approaches in parenting interventions tend to place the sole focus on the parent. However, family-centred designs that allow for both parents and children to engage together in the intervention can have several benefits. In what follows, we outline four design opportunities for parenting interventions.

#### 5.2.1 Combining physical with digital, and experiential with cognitive

Parents in our study found value in having a child-oriented experiential tool like Purrble, as well as in receiving explicit cognitive-based intervention content for themselves in the form of digital and physical supports built around it, which they could pick and choose from based on their needs and preferences. This finding highlights the potential for integrating physical, experiential tools, such as Purrble, into parenting interventions to augment opportunities for parents and children to develop the target skills in the context of their daily interactions. The experiential tool can then be complemented with explicit cognitive-based supports to tailor the intervention content to individual family’s needs, strengths, and goals. Recent HCI work on designing technology-enabled emotion regulation interventions shows that such approaches including both ‘didactic’ (i.e., cognitive) and experiential design components, as well as components that support both ‘offline’ (such as intervention support in a parenting programme or an online module) and ‘on-the-spot’ (i.e., situated) learning have so far been under-represented in the HCI literature[110].

The interactivity of the particular experiential tool used in this work, Purrble, seems to play an important role in impacting family’s practices around emotions. First, the feedback children receive when engaging with the smart toy, appears to both help to engage them with the intervention in the first place, but also help them calm down enough when needed, which can serve as the first step so that the parent can then initiate a discussion around the reasons behind the child’s behaviour or feelings. Second, since the toy embodies the emotion regulation process, it becomes associated with emotional connotations and serves as a point of reference around emotion regulation in families, creating natural opportunities for discussion about emotions between parents and children.

However, it remains an open question whether the interactivity is necessary, or whether a similar, non-interactive object which is associated with a similar narrative (i.e., a small creature which needs help to calm down in the particular context of this intervention targeting emotion-related practices) and the child becomes attached to could have similar impacts. This may be particularly relevant for young children who are inclined towards imaginative play and tend to form bonds with objects like soft toys. Similarly, it would be interesting to explore if and to what extent an interactive, but not physical object with similar qualities and associated narrative could have similar impacts (e.g., could a Tamagotchi type of digital character create similar opportunities to Purrble?). Future work should explore such questions which could have important implications for the design and scalability of such interventions.

#### 5.2.2 Building child-led elements into the intervention to engage caregivers and reduce their burden

Designing additional child-oriented supports to actively involve the child in the intervention could be another way of addressing some of the challenges of engaging parents with the intervention. Prior work in family informatics has shown how designing systems that enable children to track their own activity can both be empowering for children, but also reduce some of the burden currently placed on parents to support their child’s tracking [87]. This is in line with what some parents in our study shared around potentially turning some of the activities we had envisioned as joint parent-child activities into child-only activities in order to reduce their workload. While this could potentially limit the intended impact on parent-child interactions around emotions, it also opens up exciting possibilities and interesting design questions around which parts of the intervention could be designed as child-oriented.

To create a more natural and organic interaction between parents and an intervention, designers could consider designing parts that leverage the child’s agency to engage the parent, rather than relying solely on the parent’s initiative. This approach may lead to higher engagement from parents, as they are more likely to participate if they perceive the intervention as quality time spent with their child, rather than an additional responsibility. For instance, prior work on family physical activity tracking has highlighted the positive impact of turning tracking into a fun, family practice through the use of ‘family activity challenges’ not only on promoting family wellness, but also on enabling bonding moments between parents and children [44, 87, 88, 96, 97, 99]. We envision that family-centred, digitally-mediated parenting interventions could employ similar designs, such as setting up challenges for parents and children to either complete together, or complete separately and then compare. For example, a challenge could involve asking them to share their current mood with each other, as a way of facilitating the expression of emotions between parents and children. Future work should involve children in co-design to explore such possibilities.

#### 5.2.3 Designing for flexible and customisable support

Perhaps most importantly, our data underscore the importance of designing for flexible and customisable support that caters to the unique needs and situations of individual families. This finding aligns with prior research on digital mental health interventions, which suggests that intervention design in this context cannot be one-size-fits-all [8, 12, 73]. Parents in our study had varying levels of interest and motivation to invest effort in the intervention, as well as different needs in terms of the desired level of support and scaffolding. Moreover, even though parenting interventions might have an overarching clinical target, parents have different needs, strengths, and goals, and are on different points in their parenting journey, as also supported by our findings. Therefore, it should be taken into account that a successful intervention outcome might mean different things to different families in the context of their daily lives. While for one family a successful outcome would be to have more frequent and better quality parent-child discussions around emotions, for another family success may simply be the parent remaining calm more often during moments of heightened emotion, thus, avoiding having negative interactions with their child in such moments.

#### 5.2.4 Breaking down the intervention process into tailored micro-interventions

In the context of our study, while we chose the specific intervention activities depicted in the design scenarios as both important to achieve the intervention’s target outcomes and small and effortless enough for parents to engage with, this was not reflected in what many of our participants fed back to us. Parents had different needs, strengths, and goals which appeared to directly impact how they envisioned engaging with the intervention.

A related area of work that could be particularly pertinent in designing to address such differences is that on digital micro-interventions as the building blocks of a larger intervention process [3]. Micro-interventions are highly focused and typically short interventions, which are delivered in the context of one’s daily life with little burden on the individual in order to help them reach desired proximal targets [3, 39]. While in existing parenting interventions the success of the therapeutic process is determined by reaching the desired treatment target (e.g., reducing child conduct problems), a single digital micro-intervention can be just a step along the way.

We envision that a micro-intervention approach could be a potential way to aid parents’ engagement with the intervention, by breaking down the process into multiple small steps and allowing for the dynamic tailoring of the intervention content based on the particular user’s motivations, engagement, and experience of engagement. Enabling parents to select and achieve smaller and personally meaningful goals might keep them more engaged and motivated throughout the intervention process than setting bigger goals that are harder to achieve or are not necessarily personally relevant to them. For example, if a parent finds it challenging to use techniques they learned to support their child when they have a meltdown because they are too dysregulated themselves, the first micro-intervention could be to simply remember to take a few deep breaths when their child starts shouting or crying. Once they achieve that, the next micro-intervention could be to try the next step of this process next time their child has a meltdown, such as validating their feelings.

## 6 Limitations

It is important to note that the study participants were predominantly from white, middle to middle-upper class backgrounds. As a result, the findings may not be generalisable to families from different ethnic and/or socioeconomic status (SES) backgrounds. Future research should investigate these questions with families from diverse ethnic and SES backgrounds in order to gain a more comprehensive understanding of the applicability of these findings, and to determine which design approaches would be acceptable to these families. Furthermore, not collecting children’s perspectives on the scenario probes is another important limitation of this study that needs to be addressed in future work. Children may have unique views, needs, and goals in regards to the use of such an intervention, which may differ from those of their parents. Collecting children’s perspectives on the proposed design approaches and involving them in co-design work could provide valuable insights for the development of technologies that support parent-child interaction around emotions in the family context.

## 7 Conclusion

In this paper, we explored the potential of designing socio-technical systems that would affect complex parent-child social practices in situ. Our specific focus was on supporting the parent’s role in child emotion regulation development, which is a key process affecting child well-being; and drew on prior CSCW and HCI work on a novel socially assistive robot called Purrble, which provides child-oriented emotion regulation support. The two-step research process was focused on understanding how emergent interactions around Purrble can be augmented with parent-child and parent-oriented digital supports. In particular, we first proposed 4 theory-driven design directions, each targeting a different potential parenting support practice and each being instantiated in a simple scenario that could be presented to parents; and then explored the underlying interaction design assumptions with 22 parents as part of co-design workshops. Our findings showcase the potential of designing socio-technical systems to impact and scaffold complex parent-child interactions, and suggest a number of plausible directions for future research that can be of interest to both HCI and psychology communities.

## Figures and Tables

**Fig. 3:**
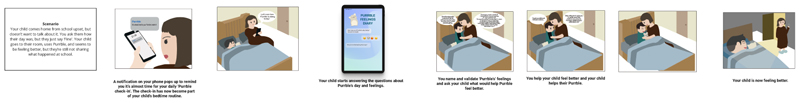
Storyboard for scenario 1

**Fig. 4:**
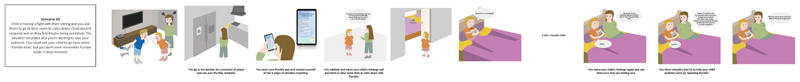
Storyboard for scenario 2

**Fig. 5:**
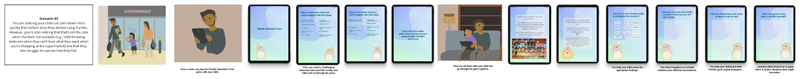
Storyboard for scenario 3

**Fig. 6:**
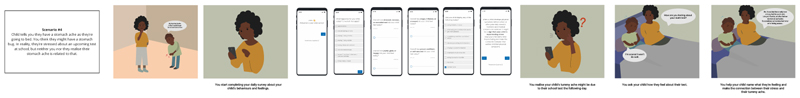
Storyboard for scenario 4
